# The reliability and quality of short videos as health information of guidance for bowel sounds: a cross-sectional study

**DOI:** 10.3389/fpubh.2025.1696018

**Published:** 2025-11-18

**Authors:** Zhang Jin, Chen Zun, Shan Liang, Liu Sida, Liu Dong, Xue Fei, Yue Qingfang, Wang Yuting, Zhang Jinping, Wu Yuling, Chen Bopeng, Duan Xianglong

**Affiliations:** 1 Department of Clinical Nutrition, Shaanxi Provincial People's Hospital, Xi'an, China; 2 Second Department of General Surgery, Shaanxi Provincial People's Hospital, Xi'an, China; 3 Second Department of General Surgery, Third Affiliated Hospital of Xi'an Jiaotong University, Xi'an, China; 4 Department of Medical Service, Shaanxi Provincial People's Hospital, Xi'an, China; 5 Department of Oncology, Shaanxi Provincial People's Hospital, Xi'an, China; 6 Shaanxi Engineering Research Center of Medical Polymer Materials, Shaanxi Provincial People's Hospital, Xi'an, China; 7 Department of Scientific Research, Shaanxi Provincial People's Hospital, Xi'an, China; 8 Department of Transplant Urology, Shaanxi Provincial People's Hospital, Xi'an, China; 9 Shaanxi International Science and Technology Cooperation Base for Clinical Medicine, Xi'an, China; 10 Institute of Medical Research, Northwestern Polytechnical University, Xi'an, China

**Keywords:** bowel sounds, information quality, social media, video platforms, clinical nutritionists

## Abstract

**Background:**

Bowel sounds are a valuable indicator of monitoring and reflecting intestinal motility. Information about bowel sounds is significant for assessing physical condition. Short video-sharing platforms facilitate such information but must be validated regarding the quality and reliability of the content.

**Objective:**

This study aimed to assess the reliability and quality of bowel sounds-related information available on Chinese short video-sharing platforms.

**Methods:**

A total of 132 video samples were collected on the three most popular Chinese video sharing platforms: TikTok, Bilibili, and WeChat. Each video was assessed by two independent physicians in terms of content comprehensiveness, quality (using the Global Quality Score) and reliability (using the DISCERN tool). Furthermore, comparisons were made across different video sources.

**Results:**

Out of 132 videos analyzed, 78 (59.09%) were uploaded by medical professionals, including gastroenterologists, non-gastroenterologists, and clinical nutritionists, while 54 (40.91%) were shared by non-medical professionals such as science bloggers, nonprofit organizations and patients. Gastroenterologists-uploaded videos received the highest engagement, with median likes of 150 (IQR: 31–1,198), favorites of 90 (IQR: 19–412) and share of 50 (14–225). And in general, Medical professionals’videos generally showed higher engagement, particularly those by Gastroenterologists, compared to non-medical professionals. The median The GQS and modified DISCERN tool were used to assess video quality and reliability respectively, with medical professionals scoring higher on both metrics (*z* = 4.448, *p* < 0.001; *z* = 2.209, *p* < 0.05). GQS score and DISCERN score was 2 for bowel sounds videos analyzed in this study. Videos from gastroenterologists had the highest GQS scores, with a median of 3. However, the DISCERN score of gastroenterologists needs to be improved.

**Conclusion:**

The study shows that medical professionals generally provide better and more accurate results than non-professionals. Videos uploaded by clinical nutritionists offer more comprehensive health education and treatment options. To ensure public access to reliable information, it’s important to encourage medical professionals to produce the videos and also basic standards must be established.

## Introduction

In recent years, with the overall improvement in quality of life, digestive system diseases have become increasingly prevalent (1). According to statistics from the World Health Organization, gastrointestinal diseases pose a significant global health burden, with over 10 million deaths annually and approximately 20% of China’s population affected, placing the country at the forefront worldwide. Furthermore, the incidence of intestinal diseases continues to rise, adversely impacting individuals’ physical and mental well-being as well as their quality of life (2, 3). Bowel sounds (4–6), as a critical physiological signal, provide valuable insights into intestinal motility and serve as an essential diagnostic tool in clinical practice for evaluating gastroenteritis peristalsis and diagnosing gastrointestinal disorders. These sounds can be perceived subjectively by patients or detected objectively using professional intelligent stethoscopes, which are non-invasive, user-friendly, and integral to the comprehensive assessment of gastrointestinal function (7, 8).

The public is increasingly aware that abnormal bowel sounds are the primary indicator of abdominal diseases, including diarrhea, intestinal obstruction, gastric cancer, colorectal cancer, and hernias (9, 10). Early detection of abnormal bowel sounds can facilitate timely intervention for these conditions. However, there is a general lack of knowledge regarding how to identify bowel sounds and the correlation between specific types of bowel sounds and diseases. Additionally, there is a notable level of fear and anxiety surrounding bowel sounds, particularly concerning how to determine their normality. The treatment of abdominal diseases caused by abnormal bowel sounds, such as acute intestinal obstructions, necessitates prompt clinical intervention. Therefore, understanding how to identify bowel sounds and implementing standardized treatment based on their characteristics are essential for improving the diagnosis and treatment rates of abdominal diseases.

Over the past 30 years, advancements on the internet have improved access to medical consultations and health education for patients (11). In the last 5 years, the emergence of 5G technology and the widespread adoption of smartphones in China have prompted more consumers.

to utilize social media platforms, such as Bilibili, WeChat, and TikTok, for health education and to select medical resources (12, 13).

In China, short video platforms are becoming vital sources of health information and education for patients, as well as a new way for physicians to connect with them. Video-based education has been shown to effectively enhance disease knowledge among atopic dermatitis patients, as evidenced by a randomized controlled trial (14, 15). Further studies on the cost-effectiveness of digital health interventions, such as network-based consultations, point to the possibility that these methods can reduce healthcare costs.

Platforms like these provide unique advantages in disseminating health information. Video content, especially, is easier to absorb than other items (12). It may be helpful for patients with abnormal bowel sounds to determine the severity of their condition. Therefore, using social media to standardize bowel sounds communication is essential (16).

There has not yet been a sufficient assessment of the quality of bowel sounds on short video sharing platforms. It aimed to evaluate the content, reliability, and quality of videos related to bowel sounds posted on Bilibili, WeChat, and TikTok.

## Methods

### Search strategy and data collecting

All collected videos were sourced from Bilibili, WeChat, and TikTok, 3 of the most popular Chinese short-video sharing platforms. 肠鸣音 (“bowel sounds”), or 异常肠鸣音 (“abnormal bowel sounds”) or 肚子咕咕叫 (“stomach rumbling”) were used as keywords for searches before May 5^th^ 2025. Videos were excluded if they were duplicates, had no sound or poor sound quality, were for commercial purposes, were irrelevant to the topic, if the author identity could not be obtained, or if they were not in Chinese.

Videos with multiple parts were counted as a single video. Basic information was extracted from each video, including the uploader’s identity, video length, number of saves, shares, and likes. All the data was recorded in Excel (Microsoft Corporation).

### Evaluating methodologies

The completeness, quality and reliability of the videos were scored using adopted grading systems similar to those used in other video evaluation studies to divide the video content into six key categories: definition, epidemiology, risk factors, outcomes, diagnosis and treatment. The quality of the content was evaluated by two raters who scored how adequately each video addressed each type of content on a 5-grade Likert scale: 0 points (no content), 0.5 points (little content), 1 point (some content), 1.5 points (more content) and 2 points (extensive content).

The content and information quality of the videos were evaluated using the modified DISCERN tool and the Global Quality Score (GQS), respectively. DISCERN first proposed by Goobie et al. (11) in 1999, is a widely validated and applied tool to help health professionals assess the quality of health-related content in videos. The tool assesses videos primarily through five questions, which are scored either “1” or “0” according to whether the answer is “yes” or “no,” with a minimum score of 0 out of 5. GQS was developed by BERNARD in 2007 and was used by SINGH in 2012 to evaluate videos. Now, it’s another widely used tool for assessing the quality of health information in videos, with response selection based on a point scale from 1 (poor quality) to 5 (good quality).

### Evaluation procedure

Before the search, the two raters responsible for evaluating video quality had not viewed any bowel sounds related videos. To eliminate potential biases from personal recommendation algorithms, we deleted all historical records and settings from the smartphone, applied for hosting, and logged into a new account on each video platform.

**Figure 1:**
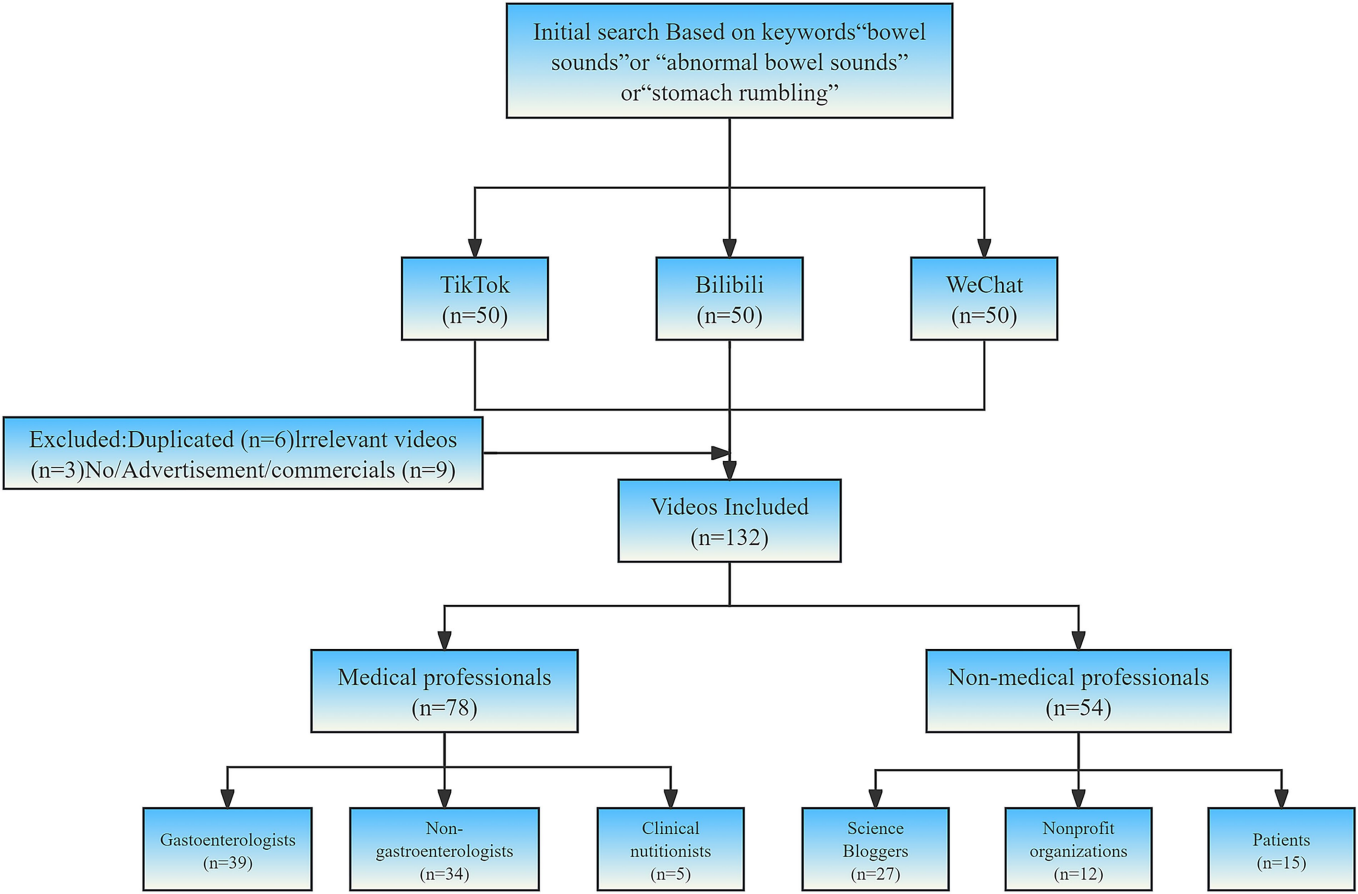
Search strategy and video screening procedure.

Based on the inclusion and exclusion criteria, 132 bowel sounds related videos were selected for further analysis (Figure 1). These videos were categorized into two groups according to the uploader’s identity: medical professionals and non-medical professionals. Within the medical professional group, video creators were further classified into three categories: Gastroenterologists, Non-gastroenterologists and Clinical nutritionists. For videos uploaded by non-medical professionals, creators were categorized into three groups: Science Bloggers, Nonprofit organizations and Patients.

Each video was rated based on several criteria. We recorded fundamental video information, including video length, the number of favorites, shares, and likes, as well as the identity of the uploader. Additionally, each video was evaluated independently by two raters using DISCERN and GQS codes to determine its content, reliability, and quality (12, 17). Prior to scoring, the raters reviewed the official scoring instructions for DISCERN and GQS. Both raters assessed each video, followed by discussion to resolve discrepancies.

### Statistical analysis

Firstly, Cohen *κ* coefficients were calculated to determine the interrater reliability. The interrater reliability for each evaluation item was greater than 0.8, indicating good interrater reliability. Secondly, SPSS 25.0 statistical software was used to analyze the data. Mean and standard deviation were calculated for normally distributed data, and median and interquartile range (IQR) were calculated for non-normally distributed data. Mann–Whitney tests were used for comparisons between two groups, while Kruskal–Wallis H tests were used for comparisons among three or more groups. The count data were expressed as a rate (%) and analyzed by chi-square (χ2) test. The correlations between different datasets were analyzed using Spearman correlation analysis. Statistical significance was set at *p* < 0.05. R software (version 4.3.0) and Origin 2021 were also used for statistical analysis and data visualization.

## Results

### General information and characteristics of the videos

As shown in Table 1, 78 of the 132 videos were shared by medical professionals (59.1%), whereas 54 were shared by non–medical professionals (40.9%). In the medical professionals, the proportion of videos uploaded by gastroenterologists (*n* = 39, 29.5%) and non-gastroenterologists (*n* = 34, 25.8%) is roughly the same, with the Clinical nutritionists uploading the least (*n* = 5, 3.8%). In contrast, in the non-medical professionals, science bloggers contribute the most (*n* = 27, 10.4%), followed by patients (*n* = 15, 11.4%), and finally nonprofit organizations (*n* = 12, 9.1%). Across all included videos, we found that videos uploaded by Medical professionals had a longer duration (median: 77, IQR49.75–241.75), higher favorites (median: 53.5, IQR16.5–336.25), more shares (median: 33.5, IQR15.5–141.75) and even more likes (median: 69, IQR26.5–386.75). Surprisingly, we found that videos uploaded by clinical nutritionists in medical professionals had a longer duration (median: 137, IQR 93.5–313.5) but received the least likes (median: 36, IQR 20–41.5) than videos from others in medical professionals.

**Table 1:** Characteristics of the videos across sources.

Source (Description)	Length of video (seconds) median (IQR)	Favorites median (IQR)	Share median (IQR)	Likes median (IQR)	Videos (%)
Medical professionals					
Gastroenterologists	72 (41–220)	90 (19–412)	50 (14–225)	150 (31–1,198)	39 (29.5)
Non-gastroenterologists	81.5 (56.75–274.5)	27 (11.75–334)	30 (12–102.75)	60 (24.5–302.25)	34 (25.8)
Clinical nutritionists	137 (93.5–313.5)	31 (17–49)	43 (26–52.5)	36 (20–41.5)	5 (3.8)
Total	77 (49.75–241.75)	53.5 (16.5–336.25)	33.5 (15.5–141.75)	69 (26.5–386.75)	78 (59.1%)
Non-medical professionals					
Science bloggers	44 (27–68)	32 (15–131)	18 (18–62)	29 (12–67)	27 (20.4)
Nonprofit organizations	67.5 (55–79.5)	47 (10–121.75)	10 (35–38.5)	8 (3.25–62)	12 (9.1)
Patients	58 (38–80)	72 (15–109)	29 (12–81)	21 (8–43)	15 (11.4)
Total	58 (29.5–78.25)	39 (15–110.5)	24 (14.25–59)	23.5 (7.75–65.5)	54 (40.9%)

### Video content

**Figure 2:**
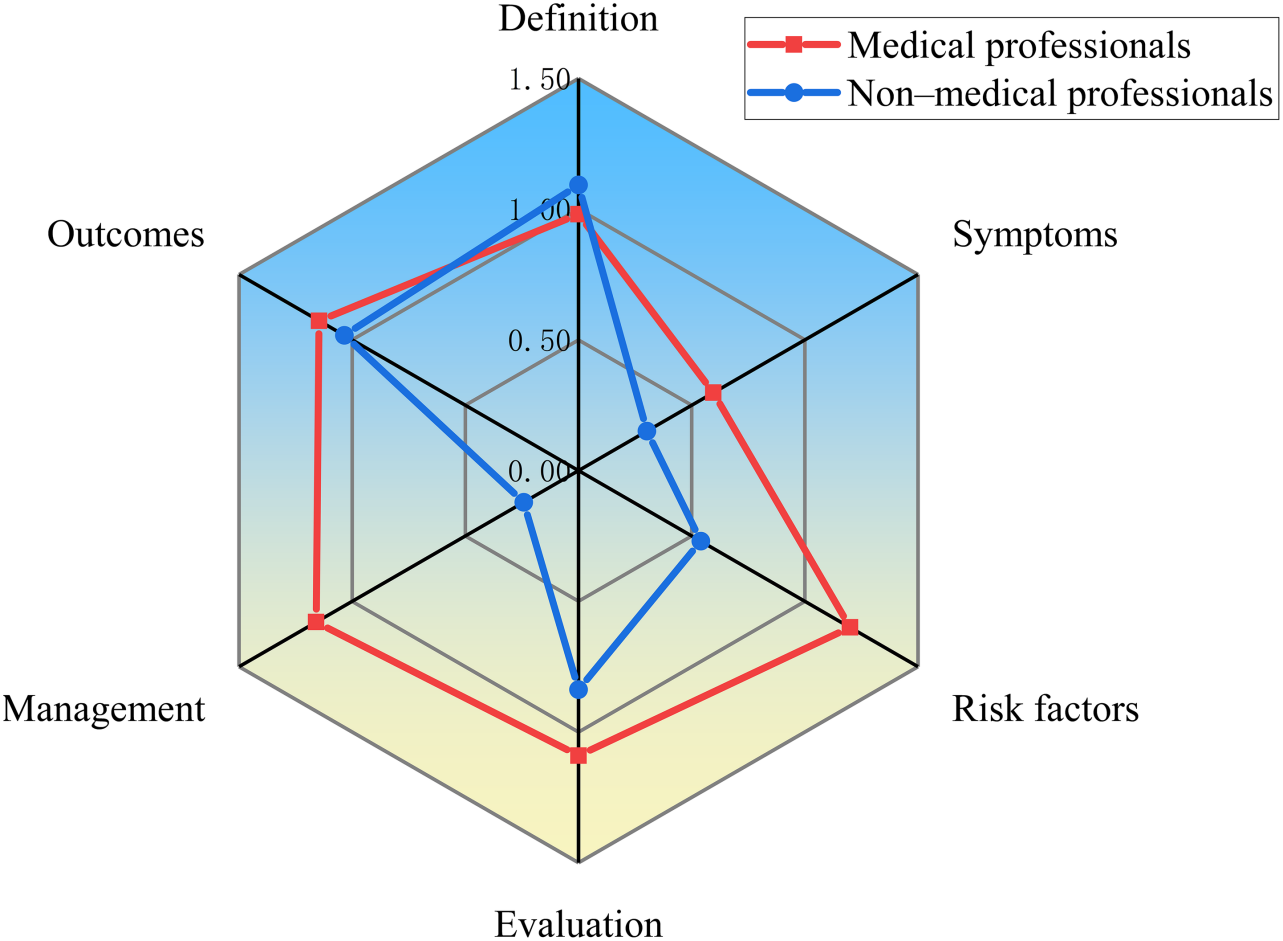
Comparison of content comprehensiveness between medical professionals and non-medical professionals.

**Figure 3:**
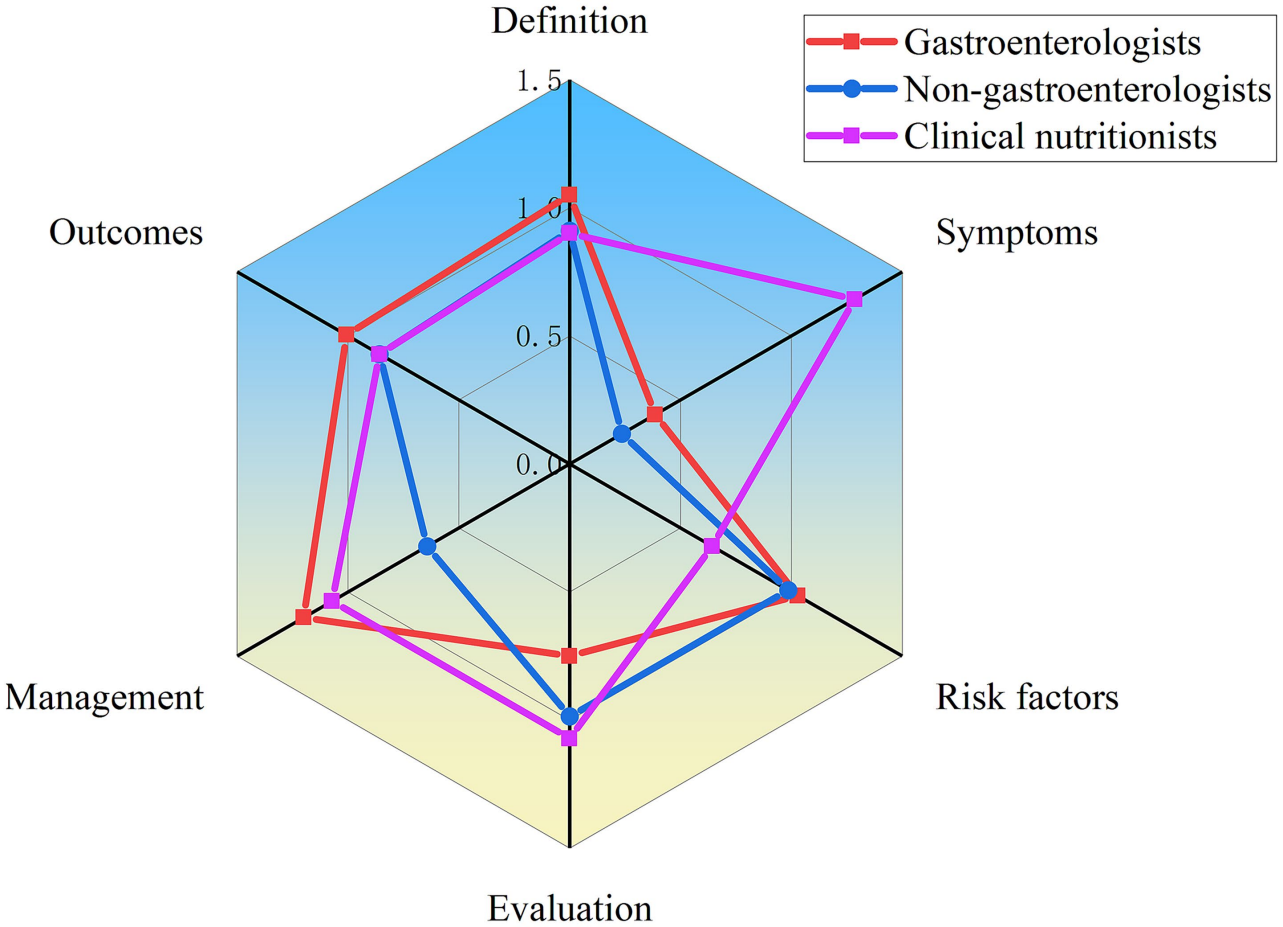
Comparison of content comprehensiveness between different medical professionals.

**Figure 4:**
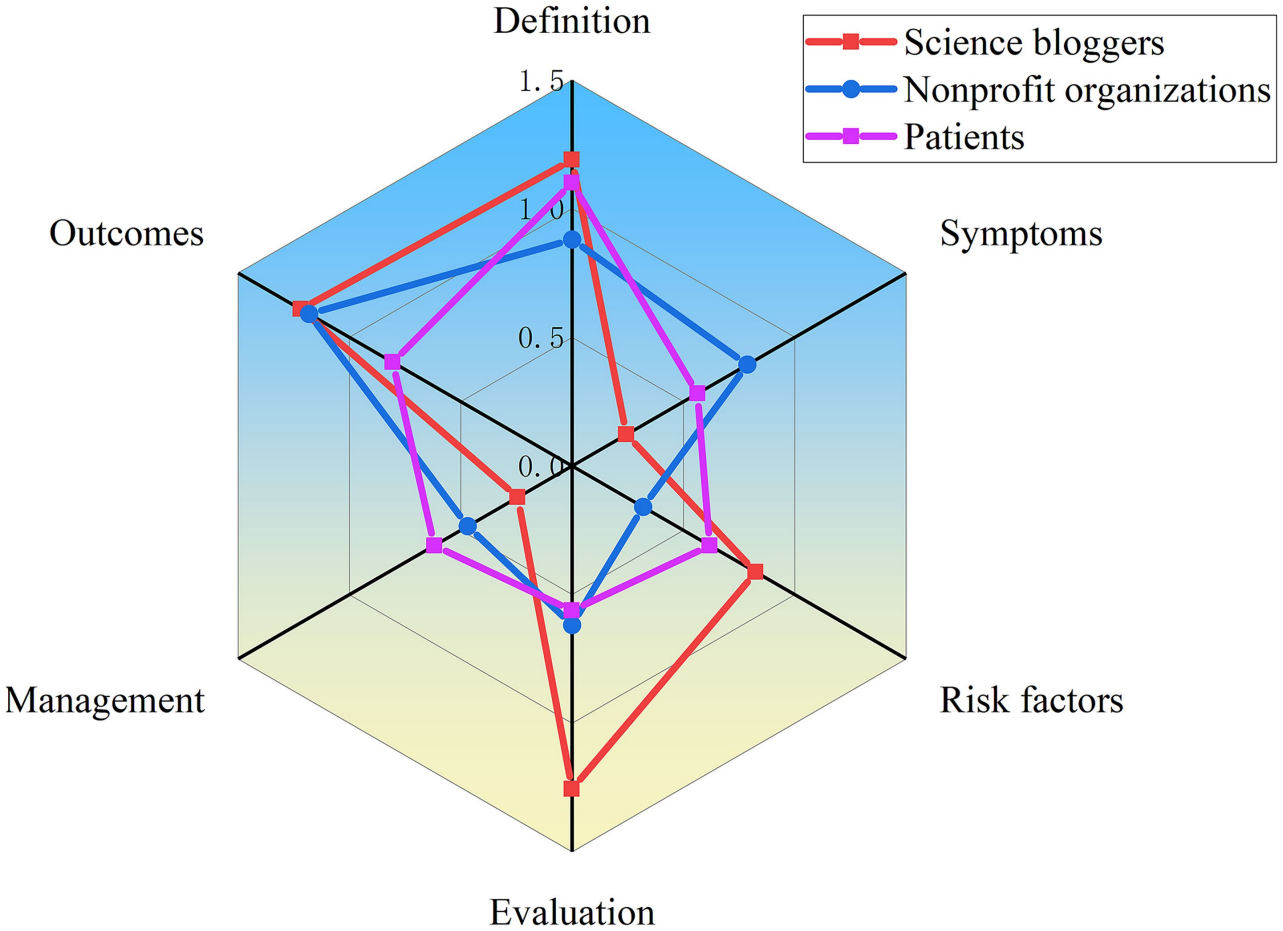
Comparison of content comprehensiveness between different non-medical professionals.

The content comprehensiveness of each video was evaluated according to the six main aspects and common current bowel sounds issues described in the Methods section. As shows in Table 2, 50% of the videos did not mention or rarely mentioned symptoms for bowel sounds (classified as “no” or “few” content). But most video content focused on the definition, risk factors, evaluation and outcomes of bowel sounds, accounting for 71.2, 80.3, 76.4, and 76.5% (classified as “some,” “most,” or “extensive” content), respectively. We then compared content comprehensiveness across different video sources. As illustrated in Figure 2, videos created by medical professionals had a higher overall content score than those created by non-medical professionals, except for definitions. Following an evaluation of the two groups’ content, we found that clinical nutritionists offered more comprehensive health education and treatment options than other medical professionals (Figure 3). Non-medical professionals tended to focus more on definitions, evaluations, and outcomes in videos from science bloggers, while patients and non-profit organizations focused more on symptoms and management (Figure 4).

**Table 2:** Completeness of video content.

Video content	Definition *n* (%)	Symptoms *n* (%)	Risk factors *n* (%)	Evaluation *n* (%)	Management *n* (%)	Outcomes *n* (%)
No content (0 points)	10 (7.6)	18 (13.6)	10 (6.8)	11 (8.3)	12 (9.1)	9 (6.8)
Few content (0.5 points)	28 (21.2)	48 (36.4)	21 (12.9)	20 (15.2)	38 (28.8)	22 (16.7)
Some content (1 point)	56 (42.4)	37 (28.0)	48 (53.8)	56 (42.4)	35 (26.5)	50 (37.9)
Most content (1.5 points)	21 (15.9)	21 (15.9)	43 (19.7)	32 (24.2)	41 (31.1)	32 (24.2)
Extensive content (2 points)	17 (12.9)	8 (6.1)	10 (6.8)	13 (9.8)	6 (4.5)	19 (14.4)

### Information quality and reliability

**Figure 5:**
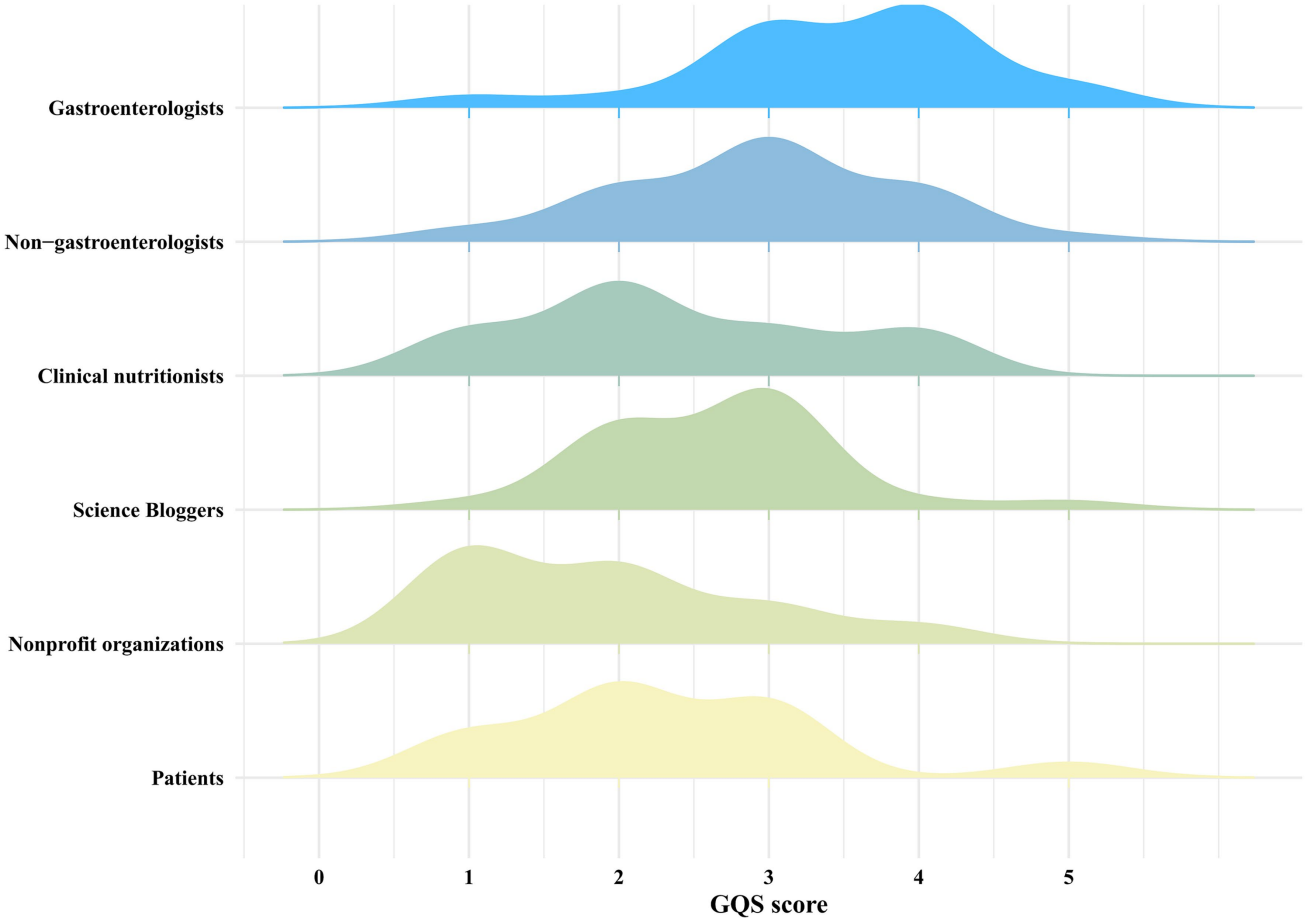
The overall distribution of GQS scores among different sources.

**Figure 6:**
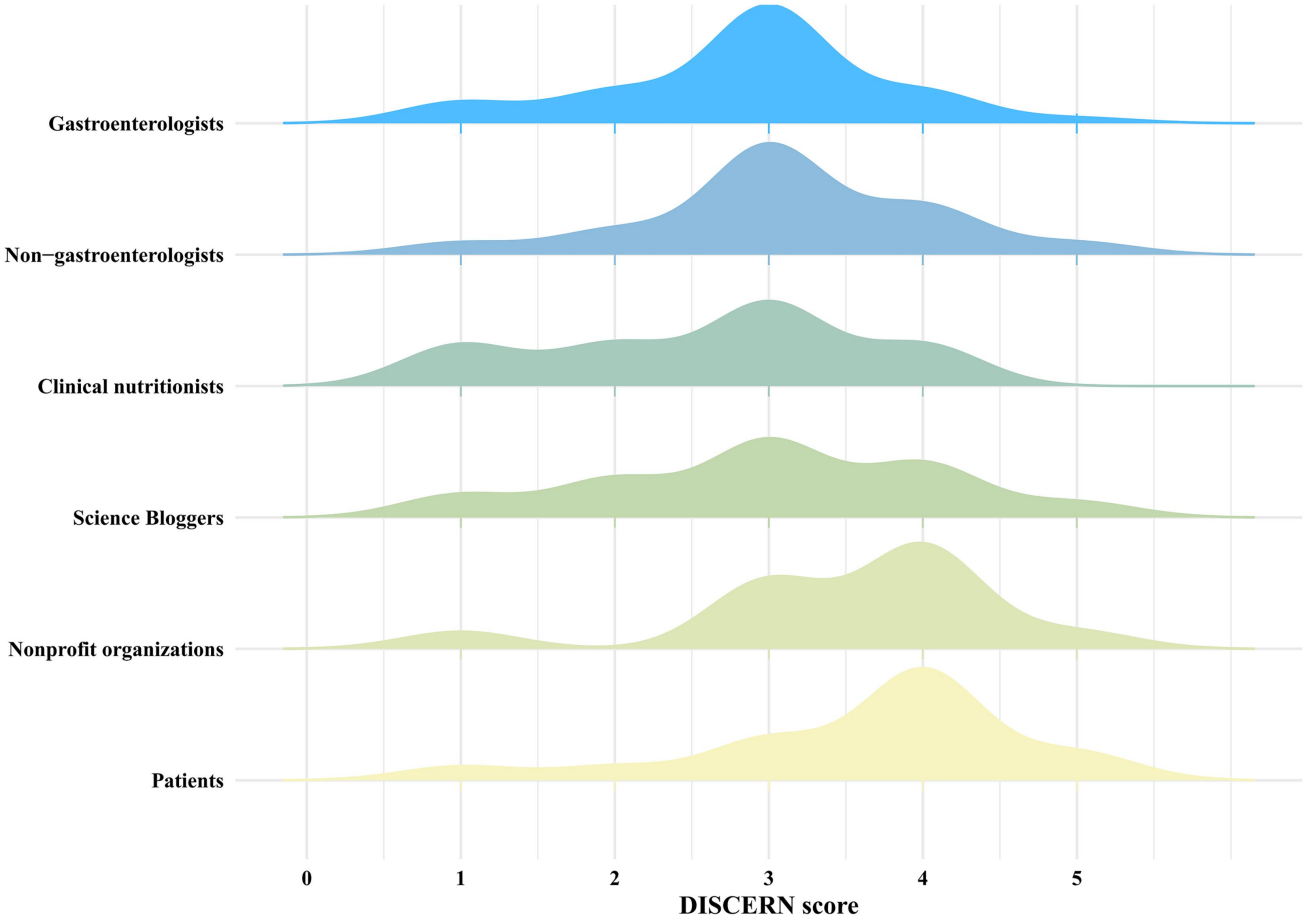
The overall distribution of DISCERN scores among different sources.

We first use GQS to assess the overall quality of each video. As shown in Table 3, videos created by medical professionals scored higher on the GQS than those from non-medical professionals (*z* = 4.448, *p* < 0.001). Further analysis indicated that videos by gastroenterologists had higher quality scores than those from others in medical professionals (χ^2^ = 35.462, *p* < 0.001) (Figure 5). And videos by Science Bloggers had higher quality scores than those from others in non-medical professionals (χ^2^ = 32.111, *p* < 0.001) (Figure 5). The DISCERN was used to assess video reliability, consistent with the GQS findings, reliability scores for videos by medical professionals were higher than those by non-medical professionals (*z* = 2.209, *p* < 0.05). Additional analysis revealed that videos by gastroenterologists had higher quality scores than those from others in medical professionals (χ^2^ = 60.974, *p* < 0.001). As with GQS, videos by Science Bloggers had higher scores than those from others in non-medical professionals (χ^2^ = 21.556, *p* < 0.001) (Figure 6).

**Table 3:** Comparison of videos from different types of uploaders.

Source	GQS scores median (IQR)	GQS scores mean (SD)	DISCERN scores median (IQR)	DISCERN scores mean (SD)
Medical professionals				
Gastroenterologists	4 (3–4)	3.49 (0.94)	3 (2–3)	2.85 (0.90)
Non-gastroenterologists	3 (2–4)	2.94 (0.92)	3 (3–4)	3.12 (0.91)
Clinical nutritionists	2 (1.5–3.5)	2.40 (1.14)	3 (1.5–3.5)	2.60 (1.14)
Over all	3 (3–4)	3.18 (0.99)	3 (3–3)	2.95 (0.92)
Non-medical professionals				
Science blogger	3 (2–3)	2.67 (0.78)	3 (2–4)	3 (1.11)
Nonprofit organizations	1 (2–2.75)	1.92 (0.99)	4 (3–4)	3.5 (1.00)
Patients	2 (2–2)	2.33 (1.04)	4 (3–4)	3.6 (1.06)
Over all	2 (2–3)	2.41 (0.94)	3 (3–4)	3.28 (1.09)

## Discussion

It is important to recognize that bowel sounds vary based on the dynamic changes in intestinal motility (18–20). As a result of pathological conditions that affect the intestines, bowel sounds can be categorized into three distinct types:hyperactive (exceeding 10 sounds per minute), markedly hyperactive (exceeding 15 sounds per minute), and hypoactive or absent. Normal bowel sounds occur about four to five times per minute in healthy individuals. It is common for them to be more frequent postprandially and to diminish during rest. Intestinal spasms or enteritis are frequently associated with gurgling or high-pitched sounds. As a result, high-pitched, tinkling, or metallic sounds can signal intestinal obstruction. Artificial intelligence (AI) based on bowel sound monitoring platforms now provide disease alerts for both healthy individuals and patients through real-time bowel sound auscultation (21–23). Consequently, both patient populations and physicians are increasingly aware of this basic physical sign.

Research indicates that patients are increasingly relying on social media for medical information (24–28). This study conducted a systematic quality and credibility assessment of content related to bowel sounds on mainstream short video platforms, confirming that these platforms are effective channels for disseminating information in the field of bowel sounds. Analysis revealed significant correlations between video duration and multiple indicators. These findings provide a basis for optimising platform content. The study notes that while short videos are effective at quickly capturing attention, they have limitations in conveying comprehensive, high-quality information; conversely, while long videos offer educational and informational advantages, they often result in lower levels of interactive engagement. Therefore, content creators must strike a balance between video duration, interactive metrics, and content quality assurance.

This study further revealed that, with the exception of definition-focused videos, content produced by medical professionals demonstrated superior content coverage across the other five categories and achieved higher overall content scores compared to videos created by Non-medical professionals. Analysis suggests that definition-focused content tends to be inherently dry and conceptually challenging. Consequently, explanations provided by medical professionals in this category may be less easily comprehensible to lay public than those offered by the Non-medical professionals. Therefore, in future video content creation, medical professional creators need to place greater emphasis on effectively elucidating the definition of bowel sounds to enhance comprehension for the public.

This study further indicates that among medical professionals, videos created by clinical nutritionists provided the most comprehensive content, offering patients richer evaluation and treatment (29, 30). Clinical nutritionists has emerged as a highly prominent specialty in recent years, with a core mission of shifting the focus from disease treatment to prevention (28, 29). In recent years, national health authorities have established clearer development priorities for clinical nutrition physicians, emphasizing dedicated public health education to enhance public understanding of fundamental nutrition science (31–33). Consequently, the observed superiority in content coverage and quality is likely attributable to the higher volume of video production by clinical nutrition physicians and their robust specialized knowledge base (34, 35).

Among Non-medical professionals, videos produced by science bloggers provided greater informational depth regarding definitions, evaluations, and outcomes. In contrast, content created by patients and nonprofit organizations prioritized symptom-focused narratives and management strategies. This divergence likely stems from the fact that patients and nonprofit organizations concentrate more on the symptoms accompanying abnormal bowel sounds – such as abdominal distension, pain, and diarrhea enabling their content to resonate more readily with viewers through shared experiential perspectives (36–38).

For medical professionals, efforts should prioritize enhancing content comprehensibility, while lay creators must ensure information accuracy. Standardized health education videos require both scientific rigor and accessible presentation.

A synergistic model combining the clinical authority of medical professionals with the relatable communication styles of non-medical professionals maximizes the reach and impact of bowel sound-related content. This integrated approach ultimately enhances public health literacy and improves patient outcomes.

For platforms, the short videos displayed must better guide patients on the basis of ensuring scientific accuracy, thereby strengthening the dissemination of accurate health information.

Platforms that deliver short video content must ensure scientific accuracy and guide users effectively (39, 40). It is recommended to establish a dedicated health section first. Publication should only be permitted for content that has been professionally certified or strictly reviewed, and medical advertisements and false information should be strictly prohibited. The second requirement is that platforms must strictly control publication qualifications. Medical experts, licensed institutions, and reputable patients should be the only ones permitted to post, with their credibility clearly identified. It is also important to have a professional review. The health section requires qualified medical expert teams to review videos for scientific accuracy and timeliness. In addition, users can access nationally certified online hospital appointment booking services directly or others.

## Limitation

This study has several limitations that need to be pointed out. Firstly, we only included videos related to bowel sounds from the three major domestic short-video platforms TikTok, Bilibili, and WeChat, and the algorithmic and self selection bias of these platforms, particularly also with the firewall of China, which may not fully reflect the overall situation. Therefore, the research results cannot be generalized to international platforms. Secondly, the evaluation process of short videos has a certain degree of subjectivity, such as the GQS score and DISCERN score, which may lead to subjective bias affecting the research results. Finally, this study did not assess the impact of short videos on patients’ visits to the doctor or even their prognosis, and some sample sizes were relatively small. Therefore, future research should improve this study by expanding the scope of the investigation platforms and enriching the search terms.

## Conclusion

We collected 132 eligible videos from TikTok, Bilibili, and WeChat platforms for comprehensive evaluation. Our research surprisingly found that content from clinical nutritionists provided with substantially richer health education and therapeutic guidance. This superior performance is likely attributable to their higher volume of video production, robust specialized knowledge base, and deeper understanding of core requirements for effective medical short-form videos. Overall, to enhance the quality of videos related to bowel sounds, it is necessary to encourage medical professionals to produce the videos, establish basic norms for the content of such videos and a collaborative mechanism between the platform and creators to ensure that the public can obtain accurate, scientific and understandable information.

## Data Availability

The raw data supporting the conclusions of this article will be made available by the authors, without undue reservation.
